# Integrated metabolomics and lipidomics analyses suggest the temperature-dependent lipid desaturation promotes aflatoxin biosynthesis in *Aspergillus flavus*

**DOI:** 10.3389/fmicb.2023.1137643

**Published:** 2023-03-31

**Authors:** Shaowen Wu, Wenjie Huang, Fenghua Wang, Xinlu Zou, Xuan Li, Chun-Ming Liu, Wenyang Zhang, Shijuan Yan

**Affiliations:** ^1^Guangdong Key Laboratory for Crop Germplasm Resources Preservation and Utilization, Agro-biological Gene Research Center, Guangdong Academy of Agricultural Sciences, Guangzhou, China; ^2^Key Laboratory of Plant Molecular Physiology, Institute of Botany, Chinese Academy of Sciences, Beijing, China

**Keywords:** *Aspergillus flavus*, aflatoxins, metabolomics, lipidomics, temperature

## Abstract

Temperature is one of the main factors affecting aflatoxin (AF) biosynthesis in *Aspergillus flavus*. Previous studies showed that AF biosynthesis is elevated in *A. flavus* at temperatures between 28°C-30°C, while it is inhibited at temperatures above 30°C. However, little is known about the metabolic mechanism underlying temperature-regulated AF biosynthesis. In this study, we integrated metabolomic and lipidomic analyses to investigate the endogenous metabolism of *A. flavus* across 6 days of mycelia growth at 28°C (optimal AF production) and 37°C (no AF production). Results showed that both metabolite and lipid profiles were significantly altered at different temperatures. In particular, metabolites involved in carbohydrate and amino acid metabolism were up-regulated at 37°C on the second day but down-regulated from days three to six. Moreover, lipidomics and targeted fatty acids analyses of mycelia samples revealed a distinct pattern of lipid species and free fatty acids desaturation. High degrees of polyunsaturation of most lipid species at 28°C were positively correlated with AF production. These results provide new insights into the underlying metabolic changes in *A. flavus* under temperature stress.

## 1. Introduction

Aflatoxins (AFs) are highly toxic secondary metabolites produced by certain fungal species, such as *Aspergillus flavus* and *Aspergillus parasiticus*. AF-producing fungal species are distributed worldwide, especially in warm and humid regions. Climate change can significantly affect AFs production on many crops, posing a significant challenge to the safety of staple food commodities worldwide (Bai et al., 2015; Medina et al., 2017a). Several crops, such as corn, rice, wheat, peanuts, and cotton, are susceptible to *Aspergillus* species before and after harvest, leading to AF contamination (Caceres et al., 2020). AFs are harmful to the liver, kidneys, heart, brain, and nervous system in humans and livestock and may cause immunosuppression and carcinogenesis (Han et al., 2019). In addition, global economic losses from AF contamination are estimated to be in the hundreds of millions of dollars, with corn and peanuts being the most severely affected crops (Tian et al., 2021).

Environmental factors such as nutrient sources, light, pH, drought, temperature, and oxidative stress, affect the growth of AF-producing fungal species and AF biosynthesis (Kebede et al., 2012; Caceres et al., 2020). Temperatures are considered to be a primary factor affecting AF outbreaks in corn, peanut, and other crops; experiments using peanuts grown under drought stress and controlled temperature showed that high levels of AFs were produced under 29°C (Klich, 2007). Further studies found that water activity and temperature affected the expression of *aflR* and *aflS*, two AF gene cluster-specific regulators, and that temperature was the key factor involved in regulating AFB1 biosynthesis (Schmidt-Heydt et al., 2010). Previous studies have also shown that pH changes can affect the production of AF and sterigmatocystin by *Aspergillus* species, and that the PacC signaling pathway is involved in pH sensing (Tilburn et al., 1995). Early studies have found that AF production was highest at 28–30°C in *A. flavus*, but declined significantly as the ambient temperature approached the optimal growth temperature (37°C) (O'Brian et al., 2007).

A series of omics based studies have examined the mechanisms underlying temperature-regulated AF biosynthesis in *A. flavus*. Transcriptomics analysis of *A. flavus* showed that differentially expressed genes between the samples grown at 28°C and 37°C that were highly responsive to temperature changes were enriched in the “small molecule catabolic process,” “organic acid catabolic process,” “carboxylic acid catabolic process,” “fatty acid metabolic process,” and “amine catabolic process” categories (Bai et al., 2015). In particular, transcriptomic studies showed that genes involved in AF biosynthesis, such as the transcriptional regulators, *aflR* and *aflS*, were down-regulated at temperatures higher than 28°C (Bai et al., 2015; Han et al., 2019; Tian et al., 2021). Proteomics analysis of *A. flavus* grown at 28°C and 37°C revealed that temperature-regulated proteins were mainly involved in translation-related pathways, metabolic pathways, and the biosynthesis of secondary metabolites (Bai et al., 2015). Tandem mass tag-based quantitative proteomic analysis of *A. flavus* grown in liquid and solid media at different temperatures showed that the regulation of AF biosynthesis is a complex process involving many factors, such as nutrient uptake, degradation of valine, leucine and isoleucine, G protein signaling pathways, and oxidative stress (Wang et al., 2019; Wu et al., 2022).

In previous studies, the effects of temperature on AF biosynthesis have been investigated from transcriptomic and proteomic perspectives. Specifically, the results have shown that the metabolism of carbohydrates, amino acids, fatty acids, and the biosynthesis of secondary metabolites in *A. flavus* were regulated by temperature. However, the transcriptomes and proteomes do not reflect changes in metabolite levels. Hence, in this study, we conducted metabolomics and lipidomics analyses of mycelia grown at 28°C and 37°C across 6 days. The metabolomic data showed that temperature significantly regulated primary metabolism in *A. flavus*. On the second day, the level of most primary metabolites was higher at 37°C than at 28°C. Subsequently, the content of metabolites declined more rapidly at 37°C with time. The lipidomics and targeted fatty acids analyses revealed distinct patterns of unsaturation in fatty acids and lipid side chains at 28°C and 37°C. These results provide new insight into the metabolic changes underlying temperature-regulated AF biosynthesis.

## 2. Materials and methods

### 2.1. Fungal strains and biochemical experiments

*A. flavus* strain A3.2890, obtained from the China General Microbiological Culture Collection Center at the Institute of Microbiology, Chinese Academy of Sciences, was used for all experiments conducted in this study (Yan et al., 2012, 2015). Sixty μL of *A. flavus* spore suspensions stored at −80°C in glycerol was pre-cultured on potato-dextrose agar plates at 37°C for 4 days. Mature spores on the surface were harvested and resuspended in sterile distilled water containing 0.05% Tween 20 (Sigma, St. Louis, United States), diluted to a series of spore densities after counting with a haemacytometer. Two mL of spore suspensions of desired density were added to 18 ml GMS liquid media, cultured on a shaker (180 rpm) at 28°C or 37°C in the dark as described (Yan et al., 2012) with an initial spore density of 0.8 × 10^6^ spores/mL. The measurements of dry weight, glucose, kojic acid, and AFs in GMS media were performed according to protocols described previously (Yan et al., 2012, 2015). The NH_4_^+^ content in GMS media was measured using a Multi N/C (2,100 S) analyzer (Avila et al., 2017).

### 2.2. Gas chromatography-tandem mass spectroscopy (GC–MS)-based metabolomic analysis

Metabolomes were analyzed for mycelial samples grown for two, three, four, five, and six days at 28^°C^ and 37^°C^, respectively. There were five biological replicates for each sample. Mycelia were lyophilized, and metabolites were extracted from mycelial samples as previously described (Yan et al., 2017). Firstly, 1 mL of extraction solvent 1, including methyl tert-butyl ether and methanol (3:1, vol/vol), was added to extract mycelial samples, which contains 1,2-diheptadecanoyl-*sn*-glycero-3-phosphocholine (50 μL of a 1 mg/mL stock solution in chloroform), and ^13^C-Ribitol (1 mg/mL stock solution) added as internal standard for the LC–MS analysis of lipidomic, and GC–MS analysis of primary metabolites, respectively. Secondly, the mixed samples were incubated on an orbital shaker for 10 min and sonicated for 15 min. Then a volume of 500 ml of extraction solvent 2, including water and methanol (3:1, vol/vol), was added to the samples for phase separation. The extract was centrifuged at 23,128 g for 10 min at 4°C. A fixed volume of 150 μL of the polar phase (the lower phase) and one fixed volume of 500 μL of the unpolar phase (the upper phase) was transferred into a pre-labeled 1.5 mL microcentrifuge tube, respectively. Then the samples were dried in a SpeedVac concentrator without heating. For primary metabolite profiling, a dried 150 μL aliquot from the lower phase was derivatized using N-methyl-N-(trimethylsilyl) trifluoroacetamide and analyzed with GC–MS (7890A-5975C, Agilent Technologies Inc., Santa Clara, CA, United States) as previously described (Yan et al., 2018). One μL was taken from each sample and injected into GC–MS at 270°C in a split mode (50: 1) with helium carrier gas (> 99.999% purity) flow set to 1 ml/min and separated by a DB-35MS UI (30 m × 0.25 mm, 0.25 μm) capillary column. The temperature was isothermal for 4 min at 90°C, followed by an 8°C per min ramp up to 205°C, then held for 2 min, and finally ramped up at a rate of 15°C per min to 310°C, held for 2 min. The transfer line temperature was set to 300°C, and the ion source temperature was set to 230°C. The mass range analyzed was from m/z 85 to 700. One dried 500 μl aliquot of the unpolar phase in each sample was analyzed using LC–MS for further lipidomics study.

### 2.3. Targeted fatty acids analysis using GC–MS

Mycelia were lyophilized and extracted by ultrasonication for 30 min with 1.5 mL mixed solvents, including chloroform and methanol (2:1, v/v). After the centrifugation at 14,000 rpm for 10 min, 1 mL of supernatant was transferred to a tube with 200 μL 0.75% (m/v) KCl solution, vortexed for 15 s, centrifuged at 14,000 rpm for 10 min. Then 400 μL chloroform phase was transferred to a new glass vial for methylation using 2.0 ml sulfuric acid and methanol solution (5:95, v/v) at an 85°C water bath for 1.5 h. After cooling to room temperature, 1 mL water and 1 mL hexane were added to the mixture immediately, shaken, and centrifuged at 3,000 rpm for 10 min, then 200 μL extract from the n-hexane phase was injected into a GC–MS instrument (7890A-5975C, Agilent Technologies Inc., Santa Clara, CA, United States) set at 340°C in split mode (50: 1) with the helium carrier gas (> 99.999% purity) flux set at 1 mL/min as previously described (Yan et al., 2018), and separated by a DB-5MS UI column (30 m in length; 250 μm internal diameter, 0.25 μm film thickness; Varian, United States). The temperature was held isothermally for 3 min at 100°C, followed by a 4°C per min ramp up to 240°C, then held for 20 min.

### 2.4. Liquid chromatography (LC)-MS-based lipidomic analysis

Lipidomes were analyzed for the same mycelial samples grown for two, three, four, five, and six days at 28°C and 37°C, respectively. There were five biological replicates for each sample. A dried aliquot from the upper phase of the system described in the GC–MS-based metabolomics section was resuspended in 200 μL UPLC-grade ACN:2-propanol: dichloromethane (1:1:1, v/v/v), then analyzed using an LC–MS system equipped with reverse-phase liquid chromatography (Dionex, Thermo-Fisher Scientific, San Jose, CA, United States) and a high-resolution Orbitrap Fusion MS system (Thermo-Fisher Scientific, San Jose, CA, United States) (Yan et al., 2021). Five μL of each sample was eluted using a CORTECS® C18 column (150 mm × 2.1 mm, 2.7 μm, Waters) with a 0.4 ml/min flow rate. The mobile phase A was water: ACN (40: 60, v/v) with 0.1% formic acid and 10 mM ammonium formate, and the mobile phase B was 2-propanol: ACN (90:10, v/v) with 0.1% formic acid and 10 mM ammonium formate. The compounds were separated by an elution gradient: 20% B was firstly maintained for 0.2 min, then linearly decreased to 60% B from 0.2 to 2 min, to 100% B from 2 to 9 min, and maintained at 100% B from 9 to 10 min, then linearly increased to 20% B from 10 to 10.5 min followed by equilibration at 20% B for 3.5 min. The spray voltage was set to 3,500 and − 3,000 V in the positive-and negative-ion modes, respectively, with the following ion-source properties: sheath gas, 45 Arbs; auxiliary gas, 10 Arbs; sweep gas, 0 Arbs; ion-transfer tube temperature, 320°C; vaporizer temp, 350°C. All FTMS data were acquired using the following conditions: detector type, orbitrap; orbitrap resolution, 120,000; scan range was set to m/z 200–1,200. The AGC was set at 5.0 e5, and the maximum injection time was set to 100 ms. RF lens was set to 60%, and the microscans was 1; data type, profile. All FTMS2 data were acquired using the following conditions: isolation mode, quadrupole; isolation window, 1 m/z; detector type, orbitrap; scan range, auto; AGC target, 5.0 e4; maximum injection time, 100 ms; microscans, 1; orbitrap resolution, 30,000; first mass, 50 m/z; data type, profile. The HCD collision energy was set to 25% with ± HCD collision energy set to 5% in positive mode, and the HCD collision energy was set to 30% with ± HCD collision energy set to 5% in negative mode. All data was acquired by the Xcalibur 4.1 software (Thermo Fisher Scientific, United States). Thermo Scientific™ LipidSearch™ 5.0 software and SIMCA-P 13.0.3 (Umetrics, Umea, Sweden) software were used for lipidome data processing and multivariate statistical analysis, respectively.

### 2.5. Metabolomic and lipidomic data analysis

The metabolomic data were analyzed as described in our previous study (Yan et al., 2018, 2021). Specifically, GC–MS raw data were first evaluated and deconvoluted using MassHunter Qualitative Analysis B.06.00 software (Agilent Technologies Inc., Santa Clara, CA, United States). The peak integration and alignment were processed using MassHunter Quantitative Analysis B.07.01 software (Agilent Technologies Inc., Santa Clara, CA, United States). For metabolite identification, the NIST mass spectral library (version 11) and an in-house mass spectral database established using authentic standards were adopted (Yan et al., 2021). LipidSearch 5.0 software (Thermo-Fisher Scientific, Tokyo, Japan) was used for peak picking, alignment of peaks among multiple samples, and identification of lipid molecular species (Yan et al., 2021). The relative contents of metabolites, fatty acids and lipid species were normalized with QC samples prior to statistical analysis.

### 2.6. Statistical analysis

Statistical analyses were performed using R ^1^ Following log_2_ fold change (Log_2_FC) calculation and analysis of variance (ANOVA), metabolites that differed significantly between experimental groups (*p* < 0.05) were compared. FDRs were calculated for multiple pair-wise comparisons to estimate significant differences of a metabolite above the *value of p* cut-off. Compounds whose levels showed significant differences (*p* and FDR < 0.05) for at least 1 day between mycelial samples grown at 28°C and 37°C and with Log_2_FC values greater than two were normalized to the mean of the samples grown at 28°C to generate heat maps (McLoughlin et al., 2018). For multivariate statistical analysis, orthogonal partial least squares discrimination analysis (OPLS-DA) of metabolomic and lipidomic data was performed using the corresponding functions in MetaboAnalystR 3.2 (Pang et al., 2020). The pathways analysis was completed with the Pathway Analysis module based on the default quantitative enrichment analysis method and the human KEGG database (Kanehisa et al., 2017). For pathway meta-analysis, the combined *p*-values were computed using the *p*-values from the 2^nd^ to 6^th^ days and the mean p method in the metap package in R. The combined enrichment ratios were the average of the enrichment ratios from the 2nd to 6th days (Pang et al., 2021).

### 2.7. Gene expression analysis by quantitative reverse transcription PCR (qRT-PCR)

Gene expression level in *A. flavus* mycelial samples was measured by qRT-PCR. There were three biological replicates per sample. Total RNA was extracted from frozen mycelial samples that had grown for two, three, four, or five days at 28^°C^ and 37^°C^, using the TaKaRa MiniBEST Plant RNA Extraction Kit (TaKaRa, Japan) following the manufacturer’s instructions. Complementary DNA (cDNA) was synthesized from 500 ng of total RNA per sample using a HiScript-II Q RT SuperMix for qPCR (+gDNA wiper) Kit (Vazyme, Biotech Co., China) following the manufacturer’s instructions. qRT-PCR was then performed using ChamQTM SYBR®qPCR Master Mix (Vazyme Biotech Co., China) in a CFX connect system (Bio-Rad, United States) and primers listed in Supplementary Table S1. The specificity of amplification was confirmed based on the melting curve. Relative gene expression was calculated using the 2^−ΔΔCt^ method (Livak and Schmittgen, 2001) with β-tubulin as the internal control.

## 3. Results

### 3.1. Active mycelial growth at 37°C and active AF production at 28°C

**Figure 1:**
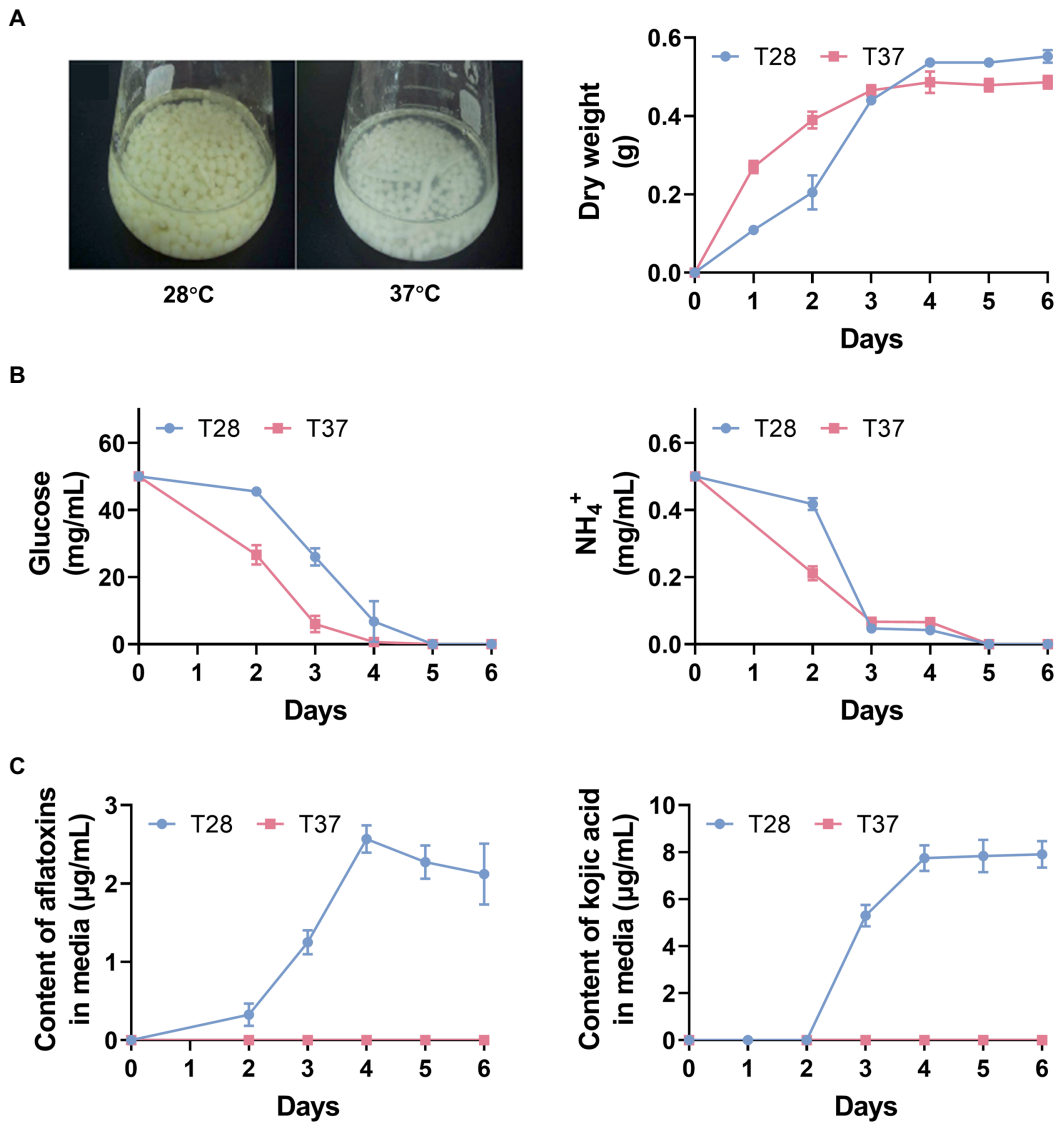
The physiological effect of temperature on *Aspergillus flavus*. **(A)** Mycelia phenotypes after 3 days of incubation and mycelia dry weight over 6 days. **(B)** Glucose and NH4^+^ contents in GMS media across 6 days. **(C)** The contents of AFs and kojic acid in GMS media across 6 days.

To examine the physiological effect of temperature on *A. flavus*, we measured mycelia growth, AF productions, and the content of glucose, NH^4+^, AFs, and kojic acid in media at 28°C and 37°C on a daily basis for 6 days. As expected, the dry weight of mycelia increased more rapidly at 37°C over the first 3 days, and glucose and NH^4+^ levels decreased more rapidly at 37°C and reached very low levels on the third day (Figures 1A,B), which was consistent with previous findings that mycelial growth is faster at 37°C, therefore carbon and nitrogen sources in the media are rapidly consumed (Liu et al., 2017; Han et al., 2019). In addition, the production of AFs and kojic acid was observed on the second and third days, respectively, and levels of them peaked on the fourth day at 28°C (Figure 1C). In contrast, negligible AFs and kojic acid production were recorded at 37°C (Figure 1C). To further confirm the low production of AFs at 37°C, we measured the AF production in both the mycelia and media of *A. flavus* strain cultured at 28°C and 37°C during a six-day period. The thin-layer chromatography (TLC) results showed that at 28°C, the production of AFs was observed in both mycelia and media samples from days three to six, while no AFs were detected at 37°C across the whole 6-day period (Supplementary Figure S1). These observations suggest that 28°C is more conducive to AF production, whereas 37°C is more conducive to mycelial growth over the first three days.

### 3.2. Amino acid and carbohydrate metabolism is activated at 37°C during The early stage of mycelial growth

**Figure 2:**
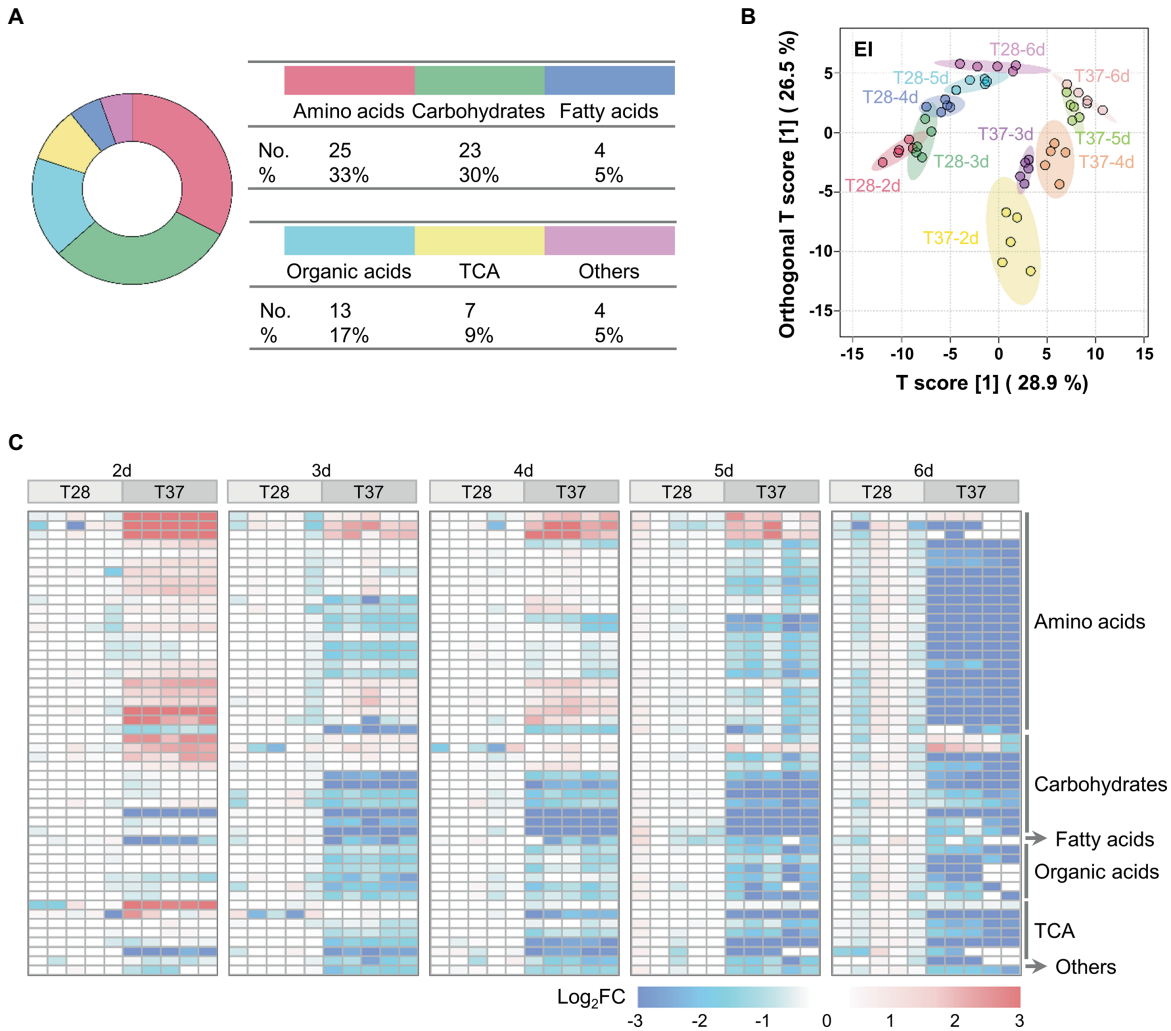
Multivariate statistical analysis of metabolomic data from *A. flavus*. **(A)** The classification and proportion of identified metabolites in *A. flavus* were obtained using GC–MS. **(B)** OPLS-DA score plots of GC–MS data for the T28 and T37 mycelia samples collected on the 2^nd^ to 6^th^ days. **(C)** Heat map showing Log_2_FC values for 50 metabolites differentially accumulated between T28 and T37 mycelia samples growth from the 2^nd^ to 6^th^ days. The relative abundance of each metabolite was normalized to the mean value from the T28 samples on different days.

To characterize and compare the metabolic profiles of mycelia samples cultured at 28°C and 37°C from two to six days (T28-2d to T28-6d and T37-2d to T37-6d), GC–MS-based metabolomic analysis was performed for mycelia samples of *A. flavus*. A total of 140 metabolites were detected (Supplementary Table S2). Among them, 76 metabolites were conclusively identified based on an in-house mass spectral database established using authentic standards, including 25 amino acids, 23 carbohydrates, four fatty acids, 13 organic acids, seven tricarboxylic acid cycle (TCA) intermediates, and four other compounds (Figure 2A). Amino acids (33%) and carbohydrates (30%) constituted the largest class of identified metabolites among the total identified components.

As shown in Figure 2B, metabolite profiles of mycelia samples collected on different days could be distinguished, although the T28-2d and T28-3d samples showed a slight overlap; samples cultured at 28°C were clearly separated from samples collected on the same day at 37°C, implying that the large metabolic difference was mediated by temperature. In addition, compared to the T28 samples, metabolites in the T37 samples collected from days two to four were relatively dispersed, which indicated that the metabolic changes occurring during the first four days of growth at 37°C were greater than those at 28°C. These results were consistent with the rapid nutrient consumption observed in T37 samples during the first four days (Figure 1A). Among the detected metabolites, the relative levels of 50 metabolites differed significantly between 28°C and 37°C, including 24 amino acids, 11 carbohydrates, one fatty acid, six organic acids, seven TCA-related metabolites, and one other compound (Figure 2C). Specifically, the relative contents of amino acids and TCA cycle intermediates were significantly up-regulated at 37°C on the second day (Figure 2C), then gradually down-regulated from days three to six.

**Figure 3:**
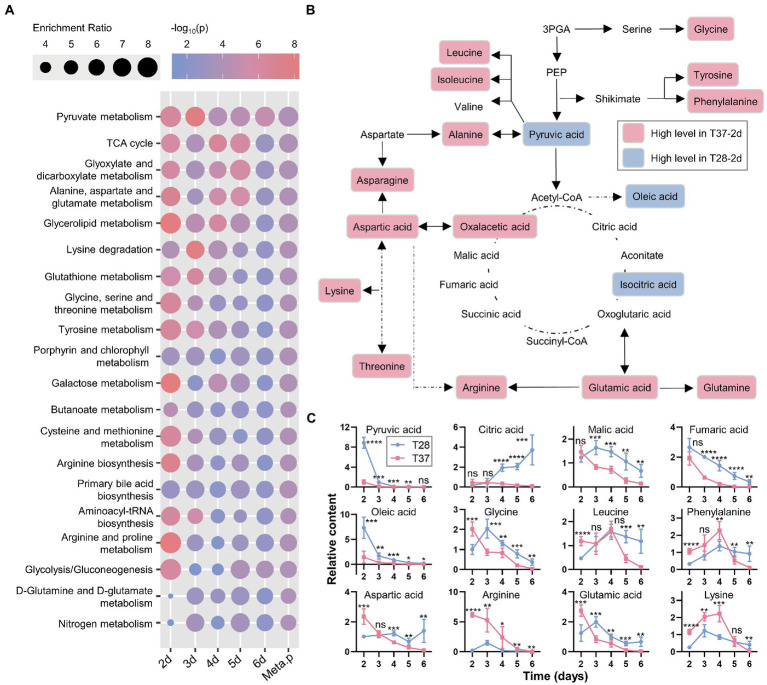
Pathway analysis and meta-analysis between the T28 and T37 mycelia samples. **(A)** Enrichment analysis and pathway meta-analysis of differentially accumulated primary metabolites. The enriched pathways were ranked using the pathway-level *p*-values (Meta.p). **(B)** An overview of metabolites involved in primary metabolism, showing the differences in metabolite levels among the T28-2d and T37-2d samples. **(C)** The relative content curves of primary metabolites. Significance was determined by the t-test. ^*^p < 0.05, ^**^p < 0.01, ^***^p < 0.001, ^****^p < 0.0001.

To examine the biological functions of the differentially accumulated metabolites, pathway analysis using the quantitative enrichment method based on their annotations and a meta-analysis on the pathway level were performed. The pathway-level *p*-values were further integrated to produce a final ranked list of enriched pathways. As shown in Figure 3A, the top five significantly enriched metabolic pathways, included pyruvate metabolism, TCA cycle, glyoxylate and dicarboxylate metabolism, alanine, aspartate and glutamate metabolism, and glycerolipid metabolism. Moreover, the significance of differences in these enrichment pathways was most significant on the second day, then gradually decreased. Most metabolites involved in the TCA cycle and amino acid metabolism were up-regulated at 37°C on the second day (Figure 3B); the relative content curves of these metabolites also peaked on the second day and decreased over time, except for pyruvic acid, fumaric acid, and oleic acid (Figure 3C). The results also suggest that metabolic activity is activated at 37°C, which is in line with the rapid mycelial growth observed at 37°C.

### 3.3. Unsaturation of lipid side chains is significantly altered by temperature

**Figure 4:**
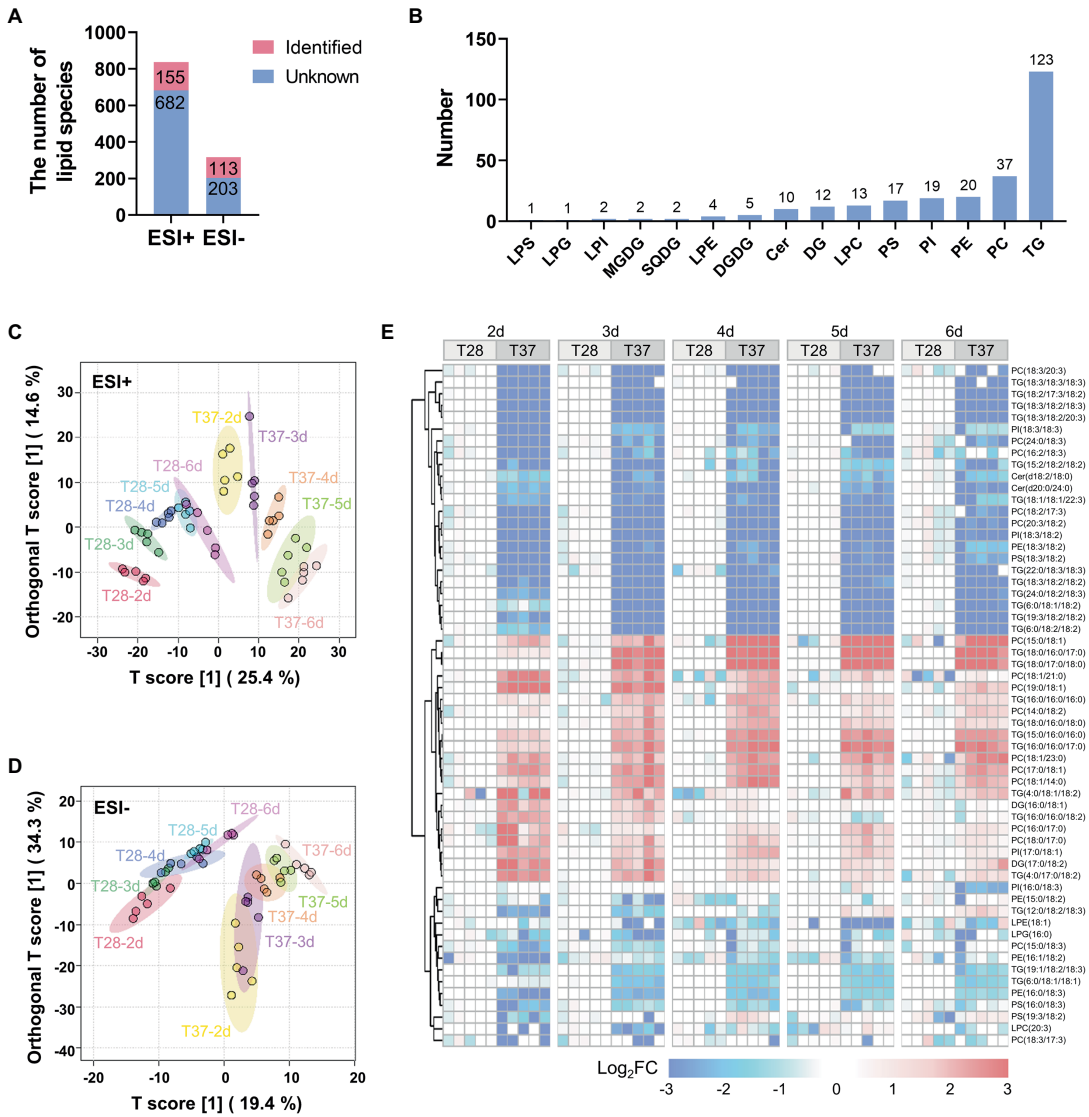
Multivariate statistical analysis of lipidomic data from *A. flavus*. **(A)** Bar diagrams showing the number of peaks detected and lipids identified by LC–MS both in the positive and negative ion modes. **(B)** The number of identified lipid species in different classes. **(C)** OPLS-DA score plots of lipidome in mycelia samples detected by LC–MS in the positive ion mode. **(D)** OPLS-DA score plots of lipidome in mycelia samples detected by LC–MS in the negative ion mode. **(E)** Heat map showing Log_2_FC values for 58 lipids differentially accumulated between the T28 and T37 mycelia samples.

Previous studies have shown that lipids and fatty acids, such as oleic acid identified in the metabolomics analysis, play important roles in regulating AF production (Priyadarshini and Tulpule, 1980). LC–MS-based lipidomic analysis was performed on the abovementioned mycelial samples to investigate the temperature-based regulation of lipids. A total of 837 and 316 lipid peaks were detected in the positive and negative ion modes, respectively (Figure 4A and Supplementary Tables S3 and S4). A total of 256 unique lipid species were identified based on their MS/MS spectra. Among them, there were 123 triglycerides (TGs), 37 phosphatidylcholines (PCs), 20 phosphatidylethanolamines (PEs), 19 phosphatidylinositols (PIs), 17 phosphatidylserines (PSs), 13 lyso-PCs (LPCs), 12 diglycerides (DGs), ten ceramides (Cers), five digalactosyldiacylglycerols (DGDGs), four lyso-PEs (LPEs), two sulfoquinovosyldiacylglycerols (SQDGs), two monogalactosyldiacylglycerols (MGDGs), two lyso-PIs (LPIs), one lysophosphatidylglycerol (LPG), and one lipopolysaccharide (LPS) (Figure 4B).

To capture differences in lipid contents between mycelia samples, OPLS-DA was performed for lipidome data obtained in positive and negative ion modes. The lipidome data obtained in the positive ion mode could be clearly distinguished between samples at different temperatures and days (Figure 4C). Similar to the metabolite profiles derived from GC–MS data, T37 samples over the growth stage were relatively dispersed compared to T28. The lipidome data obtained in the negative ion mode showed poor dispersion over the growth stage, but differences between temperatures were clearly distinguished (Figure 4D). 58 lipids, including PCs, PIs, PEs TGs, etc., significantly differentially accumulated between the T28 and T37 samples (Figure 4E). The up-and down-regulated lipids in the T37 samples contained various lipid classes, such as PCs and TGs, suggesting no significant correlation between the regulated lipid classes and temperature. However, cluster analysis of the differentially accumulated lipids revealed a unique pattern in their side chain compositions at different temperatures. Lipids with saturated fatty acid side chains (C16:0, C17:0, C18:0, etc.) were significantly up-regulated in T37 samples, while lipids with polyunsaturated fatty acid side chains (C18:2, C18:3, etc.) were significantly down-regulated. This changing pattern was similar across samples taken on days two to six. These results demonstrated that the composition of lipid side chains in *A. flavus* was greatly affected by temperature.

### 3.4. Temperature induced alterations of fatty acid unsaturation patterns are similar to that of lipid side chains.

**Figure 5:**
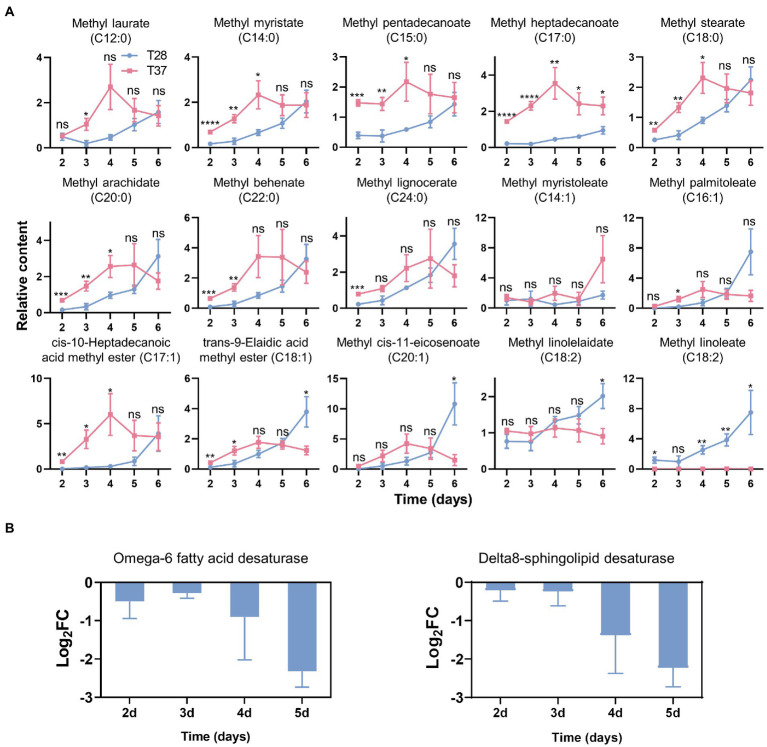
Effects of temperature on the contents of fatty acids and the fatty acid metabolism-related genes expression. **(A)** The relative contents of fatty acids quantified by GC–MS. Significance was determined by the t-test. ^*^p < 0.05, ^**^p < 0.01, ^***^p < 0.001, ^****^p < 0.0001. **(B)** the Log_2_FC of gene expression levels of fatty acid desaturases as measured *via* qRT-PCR.

To further investigate changes in endogenous fatty acids in mycelia at different temperatures, we performed a GC–MS-based fatty acid analysis, and a total of 46 fatty acids were detected in the mycelia samples (Supplementary Table S5). Among them, 17 fatty acids were conclusively identified based on an in-house mass spectral database established using authentic standards. The results showed that on the second to fifth days, the contents of most saturated fatty acids were higher in the T37 samples compared with T28 samples, except for methyl palmitate (C16:0) and methyl tridecanoate (C13:0) (Figure 5A and Supplementary Figure S2). On the second to fourth days, the contents of fatty acids containing one carbon–carbon double bond were also higher at 37°C than at 28°C. Conversely, the level of methyl linoleate (C18:2), a fatty acid containing two carbon–carbon double bonds, was significantly higher at 28°C than at 37°C over the entire growth stage. The levels of another identified fatty acid containing two carbon–carbon double bonds, methyl linolelaidate (C18:2), were slightly lower in the T28 samples on the second to third days but higher on the fourth to sixth days. These results indicate that the degree of free fatty acid unsaturation decreases with increasing mycelia growth temperature in a manner similar to the temperature-based changes observed for side-chain unsaturation of lipids.

To further examine the effects of temperature on the expression of fatty acid desaturase genes in *A. flavus*, qRT-PCR experiments were performed. The results showed that the expression of omega-6 fatty acid desaturase and Delta8-sphingolipid desaturase was down-regulated at 37°C (Figure 5B), which corroborates the increased levels of saturated fatty acids and lipids with saturated side chains observed at 37°C.

## 4. Discussion

*A. flavus* strains can produce a class of highly toxic and carcinogenic secondary metabolites, known as AFs, and the effects of climate change on their growth and AFs production are a vital point of concern. Previous studies have shown that temperature can regulate the growth and AF biosynthesis of *A. flavus*. For example, *A. flavus* produced the most AFs at 28°C and almost no AFs at 37°C (O'Brian et al., 2007; Yu et al., 2011; Bai et al., 2015; Liu et al., 2017). Most studies explored the effects of temperature on the regulatory mechanisms underlying *A. flavus* growth and AF biosynthesis from the perspective of transcriptomic and proteomic analyses, and showed that many metabolic pathways in mycelia, including carbohydrate metabolism, amino acid metabolism, and the biosynthesis of secondary metabolites, were regulated by temperature (O'Brian et al., 2007; Yu et al., 2011; Bai et al., 2015; Liu et al., 2017; Han et al., 2019; Wang et al., 2019; Tian et al., 2021). However, a detailed metabolomic description is lacking.

Previous studies on metabolomics and lipidomics of different fungi have revealed that amino acids, carbohydrates and lipids metabolism were related to the fungi’s response to environmental factors. For example, several studies showed that amino acid metabolism, carbohydrate metabolism, and the TCA cycle were involved in the fungal response to temperature stress (Puig-Castellvi et al., 2018; Zhao et al., 2020). The metabolomic analysis of *Saccharomyces cerevisiae* under low, optimal, and high temperatures showed that the level of some metabolites involved in amino acid, carbon, and sugar metabolism was high in cells grown at optimal or high temperatures, respectively, while several metabolites involved in oxidative phosphorylation and purine metabolism were also considered to be related to conditions of optimal growth (Puig-Castellvi et al., 2018). The lipidomic analysis of *S. cerevisiae* identified several classes of lipids, such as DGs, TGs, phospatidic acids (PAs), PCs, phosphatidylglycerols (PGs), PIs, PSs, inositol phosphate ceramides (PI-Cers), and found that the level of lysophospholipids, PC, and CE were high at optimal growth temperature (Puig-Castellvi et al., 2018). The transcriptomic and lipidomic analysis of *A. oryzae* revealed that lipid metabolism was involved in its response to temperature stress, and the level of triacylglycerol, PEs, and phosphoribosyl was significantly decreased at low temperatures (Jiang et al., 2022). Furthermore, the lipidomic analysis of *Neurospora crassa* and its mutant strains with deleted genes that code for the enzymes involved in the sphingolipid glucosylceramide (GlcCer) biosynthesis showed a clear knock-out specific alteration of the level of ceramide and GlcCer, while the level of PC, PE, and PI were unchanged in different strains, suggesting the specific lipid phenotype of the mutant strains (Huber et al., 2019). These results demonstrated that amino acids, carbohydrates and lipids metabolism play significant roles in the response of fungi to environmental factors.

Hence, in this work, we performed a comprehensive metabolomic, lipidomic and fatty acid analysis of *A. flavus* growth at different temperatures. Consistent with previous transcriptomic and proteomic analyses, our biochemical experiments showed that the production of AFs and kojic acid was abundant at 28°C but hard to detect at 37°C. In addition, the metabolomic analysis revealed that differentially accumulated metabolites were significantly enriched in the amino acid and carbohydrate metabolism pathways. Compared with other metabolite classes, changes in amino acids and TCA-related intermediates were more profound, with 96 and 100% of the identified amino acids and TCA-related intermediates being differentially accumulated. Previous proteome analysis showed that alanine, aspartate and glutamate metabolism, tyrosine metabolism, glycine, serine and threonine metabolism, and arginine and proline metabolism in *A. flavus* were significantly altered by temperature (Wang et al., 2019). In line with these results, our study showed that these pathways were greatly enriched between mycelia samples grown at 28°C and 37°C. Additionally, amino acid metabolism has complex effects on AF biosynthesis, and amino acids such as asparagine, aspartate, alanine, glutamate, and proline have been proposed to support AF production, while tryptophan inhibits it (Yu, 2012). The results suggested that the TCA cycle, which was found to play essential roles in regulating AF biosynthesis (Georgianna and Payne, 2009; Falade et al., 2018), as well as amino acid metabolism, was also involved in the temperature based-regulation of AF biosynthesis. In addition, carbohydrate metabolism, including pyruvate metabolism, glyoxylate and dicarboxylate metabolism, etc., connects the metabolism of other nutrients with the metabolism of glucose, increasing the production of a precursor, acetyl-CoA, for both AF and fatty acids biosynthesis. Hence, the up-regulation of pyruvate metabolism and glyoxylate and dicarboxylate metabolism on solid media was proposed to be an important reason for promoting AF production (Wang et al., 2019). Here, our results revealed that these metabolic pathways were up-regulated at 37°C on the second day, while AF production was inhibited.

Among factors reported to regulate AF biosynthesis, fatty acids and lipids have attracted much attention, as *Aspergillus* species are more likely to produce AFs after infecting crops with high oil content (Tiwari et al., 1986; Fanelli and Fabbri, 1989; Reddy et al., 1992; Severns et al., 2003). Compared to defatted seeds, unprocessed seeds promote AF production, suggesting fatty acids promote AF biosynthesis, or the ratio of carbon and nitrogen sources in the culture conditions changes the metabolism in *A. flavus* (Reddy et al., 1992; Mellon et al., 2000). In addition, the supplementation of stearic acid (C18:0), oleic acid (C18:1), or linoleic acid (C18:2) to sugar-containing media was found to inhibit AF biosynthesis (Schultz and Luedecke, 1977). However, Pyiyadarshini and Tulpule found that unsaturated fatty acids like C18:1 and C18:2 inhibit AF biosynthesis, while saturated fatty acids such as myristic acid (C14:0), palmitic acid (C16:0) and C18:0 promote it (Priyadarshini and Tulpule, 1980). Our previous studies revisited the effects of fatty acids on the production of AFs and showed that both the saturated C18:0 and the polyunsaturated linolenic acid (C18:3) promoted AF biosynthesis (Yan et al., 2015). These studies suggest that C18:1 and C18:2 inhibit, and C14:0, C16:0, and C18:3 promote the production of AFs, while the role of C18:0 in regulating AF biosynthesis is controversial between different studies (Schultz and Luedecke, 1977; Priyadarshini and Tulpule, 1980; Yan et al., 2015).

Nevertheless, these results imply that the degree of unsaturation of fatty acids may be important. Previously, Qu et al. performed the lipidomic profilings of *A. flavus* upon the treatment of antifungal compound carvacrol and found that carvacrol had a significant inhibitory effect on AFB1 production and lipids with unsaturated side chains, such as, TG (18:2/17:2/18:2), TG (18:3/18:2/18:3), TG (18:2/18:2/16:2), were significantly downregulated in a dose-dependent manner (Qu et al., 2021). In this work, we found that the degrees of unsaturation of both free fatty acids and the side chains of lipids changed in a distinct pattern dependent on temperature, with the degree of unsaturation being higher at the low temperature (28°C). In fact, *A. flavus* strains produced the most AFs at 28°C, suggesting that endogenous unsaturated fatty acids and lipids with unsaturated side chains may have roles in promoting AF biosynthesis. These findings may not be directly comparable to previous observations due to several factors, such as the difference between externally supplied and endogenous synthetic lipids or fatty acids, the limited number of fatty acids supplied in the media, and the fact that experimental conditions varied between studies.

Consistent with our findings, previous studies showed that fatty acid and lipid metabolism were significantly enriched pathways in *A. flavus* in response to temperature change (Bai et al., 2015; Medina et al., 2017b; Han et al., 2019). Medina et al. showed that the unsaturated fatty acid biosynthetic process and fatty acid beta-oxidation were enriched in *A. flavus* in response to interactions between water activity and temperature (Medina et al., 2017b). Transcriptomic analysis revealed that several genes encoding fatty acid synthase, oxygenase, and elongase, including those belonging to the AF gene cluster, *aflB*, and *aflA*, were temperature-regulated (Medina et al., 2017b; Han et al., 2019). In addition, fatty acid and lipid catabolism are linked to glucose and amino acid catabolism and help to produce more acetyl-CoA, a precursor of AF biosynthesis (Shi and Tu, 2015; Wang et al., 2019). These results suggest that lipids or fatty acids may play multiple roles in regulating AF production.

Furthermore, the oxidation of fatty acids or lipids is also an important factor affecting their functions. Lipid peroxidation was found to be involved in AF biosynthesis, and the presence of polyunsaturated fatty acids increased both the peroxidation of lipids and the production of AFs (Fanelli and Fabbri, 1989; De Luca et al., 1995; Bircan, 2006). Our previous studies showed that linolenic acid promotes AF biosynthesis while linolenic acid-derived oxylipins inhibit it (Yan et al., 2015). Additionally, lipid oxidation has previously been found to cause oxidative stress (Almeida et al., 2009). Previous studies demonstrated that oxidative stress is an essential factor in regulating AF biosynthesis, such that ROS modulators, lipid antioxidant (BHA) and dithiothreitol (DTT) significantly inhibited AFB1 production (Grintzalis et al., 2014; Caceres et al., 2020). Scarpari et al. showed that deletion of the *Aflox1* gene, coding for an Mn-dependent lipoxygenase in *A. flavus*, inhibited the synthesis of HPODEs and AFs, the inhibition of HPODEs synthesis may reduce the level of oxidative stress (Scarpari et al., 2014). Tian et al. also found that oxidative stress significantly affects lipid metabolism in *A. flavus* (Tian et al., 2021). These results suggest that fatty acid and lipid metabolism, oxidation, and oxidative stress may cooperatively regulate AF biosynthesis in *A. flavus*.

In summary, the results of metabolomic and lipidomic analyses of *A. flavus* showed that temperature significantly affects amino acid and carbohydrate metabolism in mycelia. More importantly, at 28°C, the temperature at which mycelia produce AFs, the degree of unsaturation of free fatty acids and the side chains of lipids was higher compared to 37°C. Our findings and previous results suggested a potential mechanism for how *A. flavus* produces AFs at low temperatures. That is, at low temperatures, the desaturation metabolic pathways of fatty acids and lipids are activated and result in a large amount of polyunsaturated fatty acids and lipids with polyunsaturated side chains. These unsaturated fatty acids and lipids could be oxidized (Dominguez et al., 2019), and cause oxidative stress in mycelia, thereby activating AF biosynthesis. Overall, further exploration of the regulatory mechanisms underlying fatty acid and lipid metabolism, oxidation, and oxidative stress in *A. flavus* are warranted as related findings, which may provide new strategies for AF prevention and control.

## Data availability statement

The original contributions presented in the study are included in the article/Supplementary material, further inquiries can be directed to the corresponding author.

## Author contributions

SY conceived and designed the project. WH and XL performed biochemical experiments and metabolomic and lipidomic analyses. WH, SW, FW, XZ, and WZ analyzed the metabolomic and lipidomic data. SW performed the bioinformatics analysis. SW and SY wrote the manuscript. C–ML revised the manuscript. All authors contributed to the article and approved the submitted version.

## Funding

We gratefully acknowledge the support by the Natural Science Foundation of China (31500045), the Special Fund for Scientific Innovation Strategy-Construction of High Level Academy of Agriculture Science (R2021YJ-YB1004 and R2020PY-JX019), the Natural Science Foundation of Guangdong Province (2014A030310489).

## Conflict of interest

The authors declare that the research was conducted in the absence of any commercial or financial relationships that could be construed as a potential conflict of interest.

## Publisher’s note

All claims expressed in this article are solely those of the authors and do not necessarily represent those of their affiliated organizations, or those of the publisher, the editors and the reviewers. Any product that may be evaluated in this article, or claim that may be made by its manufacturer, is not guaranteed or endorsed by the publisher.

## Footnotes

^1^ www.cran.r-project.com/

## References

[ref1] Almeida M. Ambrogini E. Han L. Manolagas S. C. Jilka R. L. (2009). Increased lipid oxidation causes oxidative stress, increased peroxisome proliferator-activated receptor-gamma expression, and diminished pro-osteogenic Wnt signaling in the skeleton. J. Biol. Chem. 284, 27438–27448. doi: 10.1074/jbc.M109.023572, PMID: 19657144 PMC2785673

[ref2] Avila C. Pelissari C. Sezerino P. H. Sgroi M. Roccaro P. Garcia J. (2017). Enhancement of total nitrogen removal through effluent recirculation and fate of PPCPs in a hybrid constructed wetland system treating urban wastewater. Sci. Total Environ. 584-585, 414–425. doi: 10.1016/j.scitotenv.2017.01.024, PMID: 28122684

[ref3] Bai Y. Wang S. Zhong H. Yang Q. Zhang F. Zhuang Z.. (2015). Integrative analyses reveal transcriptome-proteome correlation in biological pathways and secondary metabolism clusters in a. flavus in response to temperature. Sci. Rep. 5:14582. doi: 10.1038/srep14582, PMID: 26416011 PMC4586720

[ref4] Bircan C. (2006). Determination of aflatoxin contamination in olives by immunoaffinity column using high-performance liquid chromatography. J Food Quality 29, 126–138. doi: 10.1111/j.1745-4557.2006.00061.x

[ref5] Caceres I. Khoury A. A. Khoury R. E. Lorber S. Oswald I. P. Khoury A. E.. (2020). Aflatoxin biosynthesis and genetic regulation: a review. Toxins (Basel) 12:150. doi: 10.3390/toxins12030150, PMID: 32121226 PMC7150809

[ref6] De Luca C. Passi S. Fabbri A. A. Fanelli C. (1995). Ergosterol oxidation may be considered a signal for fungal growth and aflatoxin production in *aspergillus parasiticus*. Food Addit. Contam. 12, 445–450. doi: 10.1080/02652039509374328, PMID: 7664941

[ref7] Dominguez R. Pateiro M. Gagaoua M. Barba F. J. Zhang W. Lorenzo J. M. (2019). A comprehensive review on lipid oxidation in meat and meat products. Antioxidants (Basel) 8:429. doi: 10.3390/antiox8100429, PMID: 31557858 PMC6827023

[ref8] Falade T. D. O. Chrysanthopoulos P. K. Hodson M. P. Sultanbawa Y. Fletcher M. Darnell R.. (2018). Metabolites identified during varied doses of *aspergillus* species in *Zea mays* grains, and their correlation with aflatoxin levels. Toxins (Basel) 10:187. doi: 10.3390/toxins10050187, PMID: 29735944 PMC5983243

[ref9] Fanelli C. Fabbri A. A. (1989). Relationship between lipids and aflatoxin biosynthesis. Mycopathologia 107, 115–120. doi: 10.1007/BF00707547 2693964

[ref10] Georgianna D. R. Payne G. A. (2009). Genetic regulation of aflatoxin biosynthesis: from gene to genome. Fungal Genet. Biol. 46, 113–125. doi: 10.1016/j.fgb.2008.10.011 19010433

[ref11] Grintzalis K. Vernardis S. I. Klapa M. I. Georgiou C. D. (2014). Role of oxidative stress in Sclerotial differentiation and aflatoxin B1 biosynthesis in *aspergillus flavus*. Appl. Environ. Microbiol. 80, 5561–5571. doi: 10.1128/AEM.01282-14, PMID: 25002424 PMC4178614

[ref12] Han G. Zhao K. Yan X. Xiang F. Li X. Tao F. (2019). Differential regulation of mycelial growth and aflatoxin biosynthesis by *aspergillus flavus* under different temperatures as revealed by strand-specific RNA-Seq. Microbiology 8:e897. doi: 10.1002/mbo3.897, PMID: 31328901 PMC6813451

[ref13] Huber A. Oemer G. Malanovic N. Lohner K. Kovacs L. Salvenmoser W.. (2019). Membrane sphingolipids regulate the fitness and antifungal protein susceptibility of Neurospora crassa. Front. Microbiol. 10:605. doi: 10.3389/fmicb.2019.00605, PMID: 31031714 PMC6471014

[ref14] Jiang C. Ge J. He B. Zhang Z. Hu Z. Li Y.. (2022). Transcriptomic analysis reveals *aspergillus oryzae* responds to temperature stress by regulating sugar metabolism and lipid metabolism. PLoS One 17:e0274394. doi: 10.1371/journal.pone.0274394, PMID: 36094945 PMC9467314

[ref15] Kanehisa M. Furumichi M. Tanabe M. Sato Y. Morishima K. (2017). KEGG: new perspectives on genomes, pathways, diseases and drugs. Nucleic Acids Res. 45, D353–D361. doi: 10.1093/nar/gkw1092, PMID: 27899662 PMC5210567

[ref16] Kebede H. Abbas H. K. Fisher D. K. Bellaloui N. (2012). Relationship between aflatoxin contamination and physiological responses of corn plants under drought and heat stress. Toxins (Basel) 4, 1385–1403. doi: 10.3390/toxins4111385, PMID: 23202322 PMC3509714

[ref17] Klich M. A. (2007). *Aspergillus flavus*: the major producer of aflatoxin. Mol. Plant Pathol. 8, 713–722. doi: 10.1111/j.1364-3703.2007.00436.x 20507532

[ref18] Liu X. Guan X. Xing F. Lv C. Dai X. Liu Y. (2017). Effect of water activity and temperature on the growth of *aspergillus flavus*, the expression of aflatoxin biosynthetic genes and aflatoxin production in shelled peanuts. Food Control 82, 325–332. doi: 10.1016/j.foodcont.2017.07.012

[ref19] Livak K. J. Schmittgen T. D. (2001). Analysis of relative gene expression data using real-time quantitative PCR and the 2(-Delta Delta C(T)) method. Methods 25, 402–408. doi: 10.1006/meth.2001.1262 11846609

[ref20] McLoughlin F. Augustine R. C. Marshall R. S. Li F. Kirkpatrick L. D. Otegui M. S.. (2018). Maize multi-omics reveal roles for autophagic recycling in proteome remodelling and lipid turnover. Nat Plants 4, 1056–1070. doi: 10.1038/s41477-018-0299-2, PMID: 30478358

[ref21] Medina A. Akbar A. Baazeem A. Rodriguez A. Magan N. (2017a). Climate change, food security and mycotoxins: do we know enough? Fungal Biol. Rev. 31, 143–154. doi: 10.1016/j.fbr.2017.04.002

[ref22] Medina A. Gilbert M. K. Mack B. M. OBrian G. R. Rodríguez A. Bhatnagar D.. (2017b). Interactions between water activity and temperature on the *aspergillus flavus* transcriptome and aflatoxin B(1) production. Int. J. Food Microbiol. 256, 36–44. doi: 10.1016/j.ijfoodmicro.2017.05.020, PMID: 28582664

[ref23] Mellon J. E. Cotty P. J. Dowd M. K. (2000). Influence of lipids with and without other cottonseed reserve materials on aflatoxin B(1) production by *aspergillus flavus*. J. Agric. Food Chem. 48, 3611–3615. doi: 10.1021/jf0000878, PMID: 10956158

[ref24] O'Brian G. R. Georgianna D. R. Wilkinson J. R. Yu J. Abbas H. K. Bhatnagar D.. (2007). The effect of elevated temperature on gene transcription and aflatoxin biosynthesis. Mycologia 99, 232–239. doi: 10.3852/mycologia.99.2.232, PMID: 17682776

[ref25] Pang Z. Chong J. Li S. Xia J. (2020). MetaboAnalystR 3.0: toward an optimized workflow for global metabolomics. Meta 10:186. doi: 10.3390/metabo10050186, PMID: 32392884 PMC7281575

[ref26] Pang Z. Zhou G. Chong J. Xia J. (2021). Comprehensive meta-analysis of COVID-19 global metabolomics datasets. Meta 11:44. doi: 10.3390/metabo11010044, PMID: 33435351 PMC7827862

[ref27] Priyadarshini E. Tulpule P. G. (1980). Effect of free fatty acids on aflatoxin production in a synthetic medium. Food Cosmet. Toxicol. 18, 367–369. doi: 10.1016/0015-6264(80)90191-1, PMID: 7461516

[ref28] Puig-Castellvi F. Bedia C. Alfonso I. Pina B. Tauler R. (2018). Deciphering the underlying Metabolomic and Lipidomic patterns linked to thermal acclimation in *Saccharomyces cerevisiae*. J. Proteome Res. 17, 2034–2044. doi: 10.1021/acs.jproteome.7b00921, PMID: 29707950

[ref29] Qu C. Li Z. Wang X. (2021). UHPLC-HRMS-based untargeted Lipidomics reveal mechanism of antifungal activity of Carvacrol against *aspergillus flavus*. Foods 11:93. doi: 10.3390/foods11010093, PMID: 35010219 PMC8750229

[ref30] Reddy M. J. Shetty H. S. Fanelli C. Lacey J. (1992). Role of seed lipids in *aspergillus parasiticus* growth and aflatoxin production. J. Sci. Food Agr. 59, 177–181. doi: 10.1002/jsfa.2740590207

[ref31] Scarpari M. Punelli M. Scala V. Zaccaria M. Nobili C. Ludovici M.. (2014). Lipids in *aspergillus flavus*-maize interaction. Front. Microbiol. 5:74. doi: 10.3389/fmicb.2014.00074, PMID: 24578700 PMC3936598

[ref32] Schmidt-Heydt M. Rufer C. E. Abdel-Hadi A. Magan N. Geisen R. (2010). The production of aflatoxin B1 or G 1 by *aspergillus parasiticus* at various combinations of temperature and water activity is related to the ratio of *aflS* to *aflR* expression. Mycotoxin Res. 26, 241–246. doi: 10.1007/s12550-010-0062-7 23605486

[ref33] Schultz D. L. Luedecke L. O. (1977). Effect of neutral fats and fatty acids on aflatoxin production. J. Food Prot. 40, 304–308. doi: 10.4315/0362-028X-40.5.304, PMID: 30731629

[ref34] Severns D. E. Clements M. J. Lambert R. J. White D. G. (2003). Comparison of *aspergillus* ear rot and aflatoxin contamination in grain of high-oil and normal-oil corn hybrids. J. Food Prot. 66, 637–643. doi: 10.4315/0362-028X-66.4.637, PMID: 12696688

[ref35] Shi L. Tu B. P. (2015). Acetyl-CoA and the regulation of metabolism: mechanisms and consequences. Curr. Opin. Cell Biol. 33, 125–131. doi: 10.1016/j.ceb.2015.02.003, PMID: 25703630 PMC4380630

[ref36] Tian F. Lee S. Y. Woo S. Y. Choi H. Y. Heo S. Nah G.. (2021). Transcriptomic responses of *aspergillus flavus* to temperature and oxidative stresses during aflatoxin production. Sci. Rep. 11:2803. doi: 10.1038/s41598-021-82488-7, PMID: 33531617 PMC7854668

[ref37] Tilburn J. Sarkar S. Widdick D. A. Espeso E. A. Orejas M. Mungroo J.. (1995). The *aspergillus* PacC zinc finger transcription factor mediates regulation of both acid-and alkaline-expressed genes by ambient pH. EMBO J. 14, 779–790. doi: 10.1002/j.1460-2075.1995.tb07056.x, PMID: 7882981 PMC398143

[ref38] Tiwari R. P. Mittal V. Singh G. Bhalla T. C. Saini S. S. Vadehra D. V. (1986). Effect of fatty acids on aflatoxin production by *aspergillus parasiticus*. Folia Microbiol. (Praha) 31, 120–123. doi: 10.1007/BF02926829 3710316

[ref39] Wang P. Chang P.-K. Kong Q. Shan S. Wei Q. (2019). Comparison of aflatoxin production of *aspergillus flavus* at different temperatures and media: proteome analysis based on TMT. Int. J. Food Microbiol. 310:108313. doi: 10.1016/j.ijfoodmicro.2019.108313, PMID: 31476580

[ref40] Wu S. Zhang Q. Zhang W. Huang W. Kong Q. Liu Q.. (2022). Linolenic acid-derived Oxylipins inhibit aflatoxin biosynthesis in *aspergillus flavus* through activation of Imizoquin biosynthesis. J. Agric. Food Chem. 70, 15928–15944. doi: 10.1021/acs.jafc.2c06230, PMID: 36508213 PMC9785051

[ref41] Yan S. Huang W. Gao J. Fu H. Liu J. (2018). Comparative metabolomic analysis of seed metabolites associated with seed storability in rice (*Oryza sativa L*.) during natural aging. Plant Physiol. Biochem. 127, 590–598. doi: 10.1016/j.plaphy.2018.04.020, PMID: 29729608

[ref42] Yan S. Liang Y. Zhang J. Chen Z. Liu C. M. (2015). Autoxidated linolenic acid inhibits aflatoxin biosynthesis in *aspergillus flavus* via oxylipin species. Fungal Genet. Biol. 81, 229–237. doi: 10.1016/j.fgb.2014.11.005, PMID: 25498164

[ref43] Yan S. Liang Y. Zhang J. Liu C. M. (2012). *Aspergillus flavus* grown in peptone as the carbon source exhibits spore density-and peptone concentration-dependent aflatoxin biosynthesis. BMC Microbiol. 12:106. doi: 10.1186/1471-2180-12-106, PMID: 22694821 PMC3412747

[ref44] Yan S. Liu Q. Naake T. Huang W. Chen M. Kong Q.. (2021). OsGF14b modulates defense signaling pathways in rice panicle blast response. Crop J 9, 725–738. doi: 10.1016/j.cj.2020.10.007

[ref45] Yan S. Zhu C. Yu T. Huang W. Huang J. Kong Q.. (2017). Studying the differences of bacterial metabolome and microbiome in the colon between landrace and Meihua piglets. Front. Microbiol. 8:1812. doi: 10.3389/fmicb.2017.01812, PMID: 28983290 PMC5613163

[ref46] Yu J. (2012). Current understanding on aflatoxin biosynthesis and future perspective in reducing aflatoxin contamination. Toxins (Basel) 4, 1024–1057. doi: 10.3390/toxins4111024, PMID: 23202305 PMC3509697

[ref47] Yu J. Fedorova N. D. Montalbano B. G. Bhatnagar D. Cleveland T. E. Bennett J. W.. (2011). Tight control of mycotoxin biosynthesis gene expression in *aspergillus flavus* by temperature as revealed by RNA-Seq. FEMS Microbiol. Lett. 322, 145–149. doi: 10.1111/j.1574-6968.2011.02345.x, PMID: 21707733

[ref48] Zhao X. Chen M. Li Z. Zhao Y. Yang H. Zha L.. (2020). The response of Volvariella volvacea to low-temperature stress based on Metabonomics. Front. Microbiol. 11:1787. doi: 10.3389/fmicb.2020.01787, PMID: 32849404 PMC7403444

