# Supplementary material for: Integrated metabolomics and lipidomics analyses suggest the temperature-dependent lipid desaturation promotes aflatoxin biosynthesis in Aspergillus flavus
Source: Front Microbiol. 2023 Mar 31;14:1137643. doi: 10.3389/fmicb.2023.1137643 (PMC10102665; doi:10.3389/fmicb.2023.1137643)
Supplement: Supplementary file 6 [file Data_Sheet_1.docx]

***Supplementary Material***

**Integrated metabolomics and lipidomics analyses suggest the temperature-dependent lipid desaturation promotes aflatoxin biosynthesis in *Aspergillus flavus***

**Shaowen Wu^1^, Wenjie Huang^1^, Fenghua Wang^1^, Xinlu Zou^1^, Xuan Li^1^, Chun-Ming Liu^2^, Wenyang Zhang^1^, Shijuan Yan^1*^**

^1^Guangdong Key Laboratory for Crop Germplasm Resources Preservation and Utilization, Agro-biological Gene Research Center, Guangdong Academy of Agricultural Sciences, Guangzhou, 510640, China

^2^Key Laboratory of Plant Molecular Physiology, Institute of Botany, Chinese Academy of Sciences, 20 Nanxincun, Fragrant Hill, Beijing, 100093, China

*** Correspondence:** Shijuan Yan: [shijuan@agrogene.ac.cn](mailto:shijuan@agrogene.ac.cn)

1. **Supplementary Figures and Tables**
2. **Supplementary Figures**


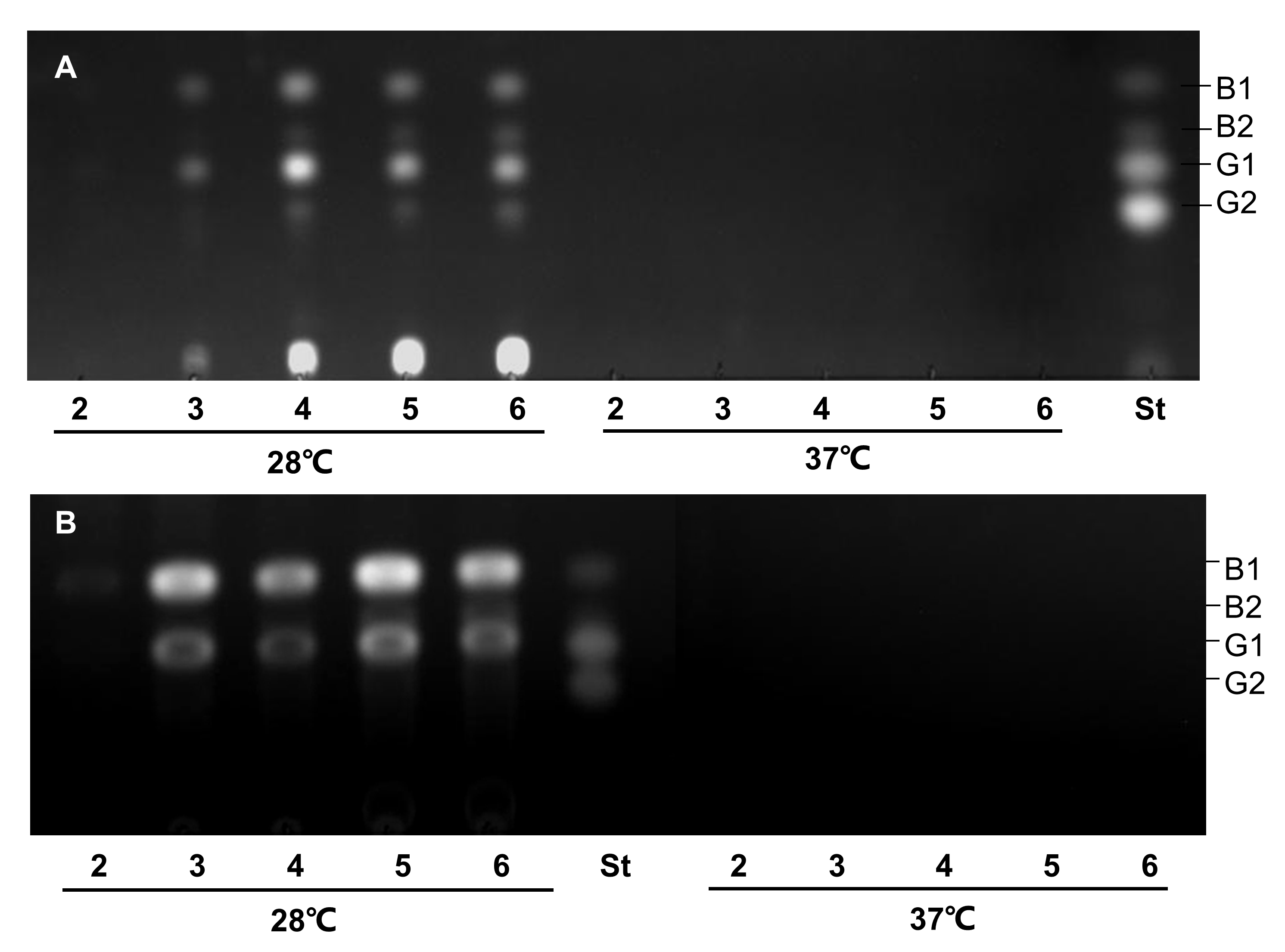


**Supplementary Figure 1. Effects of temperature on AF production in *A. flavus*.** (A) The contents of AFs in mycelia across 6 days. (B) The contents of AFs in GMS media across 6 days.


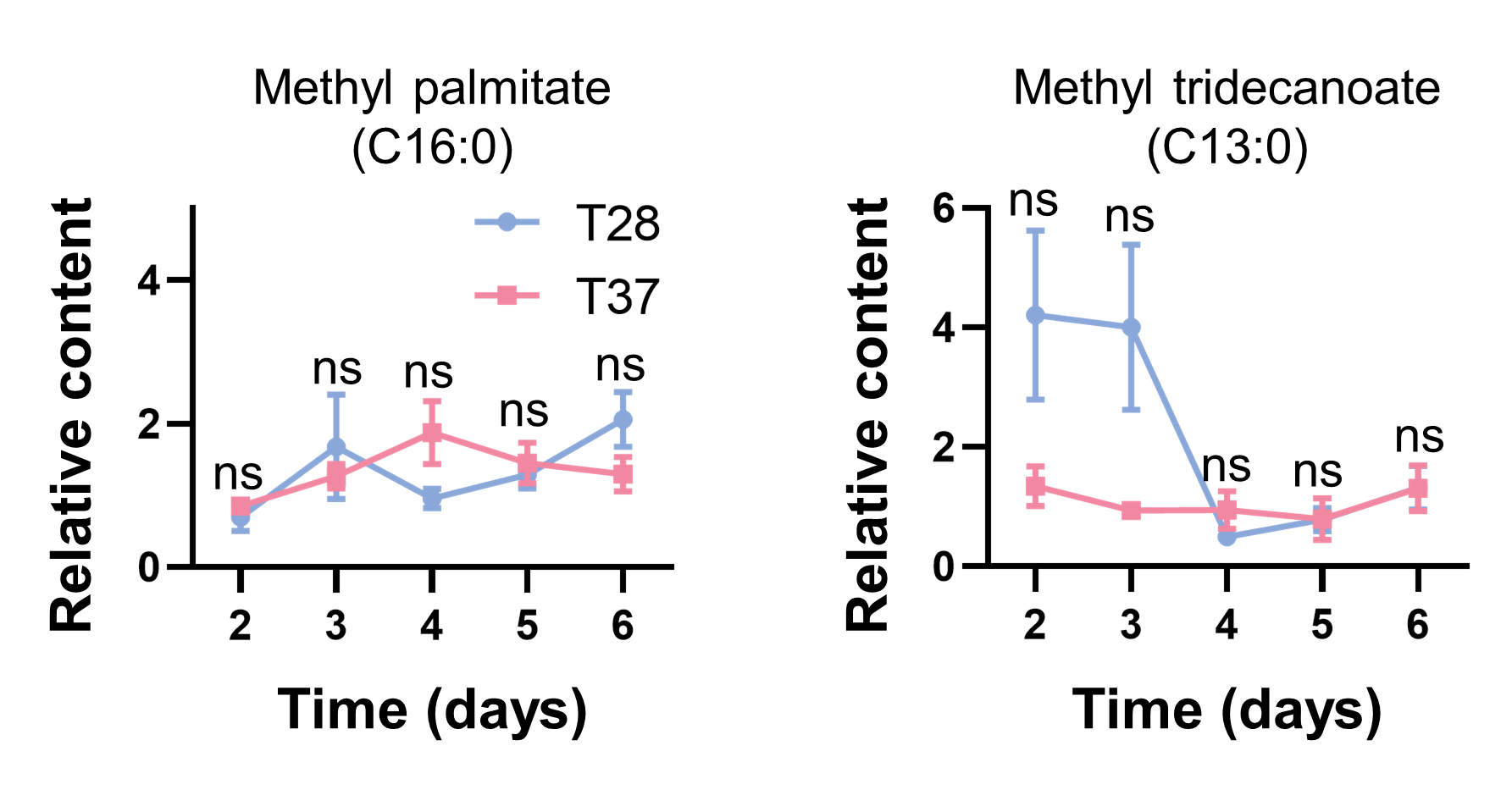


**Supplementary Figure 2.** The relative contents of fatty acids quantified by GC-MS.

1. **List of Supplementary Tables**

**Supplementary Table S1**. List of genes and primers used in this study.

**Supplementary Table S2**. The total peak information for primary metabolome in mycelia samples detected by GC-MS.

**Supplementary Table S3**. The total peak information for lipidome in mycelia samples detected by LC-MS (ESI+).

**Supplementary Table S4**. The total peak information for lipidome in mycelia samples detected by LC-MS (ESI-).

**Supplementary Table S5**. The peak information for fatty acids in mycelia samples detected by GC-MS.
